# Interaction between thermosensitive TRP channels and anoctamin 1

**DOI:** 10.1016/j.jphyss.2025.100015

**Published:** 2025-03-22

**Authors:** Yasunori Takayama

**Affiliations:** Department of Physiology, Showa University School of Medicine, 1-5-8 Hatanodai, Shinagawa-ku, Tokyo, Japan

**Keywords:** TRP channels, ANO1, Pain, Wound healing, Fluid secretion

## Abstract

Some thermosensitive transient receptor potential (TRP) channels form a protein complex with anoctamin 1 (ANO1, also called TMEM16A). TRP channels have high calcium permeability, and the calcium entering cells through TRP channel activation activates ANO1, a calcium-activated chloride channel, involved in many physiological and pathological conditions. The physiological significance of TRP channels is often mediated by their ability to activate ANO1, which controls chloride flux across the plasma membrane. This review summarizes the latest understanding on the interactions between ANO1 and thermosensitive TRP channels, including TRPV1, TRPV3, and TRPV4, which are involved in pain sensitization in primary sensory neurons, proliferation and migration of human keratinocytes, and fluid secretion such as sweat, respectively.

## Graphical Abstract

**Figure:**
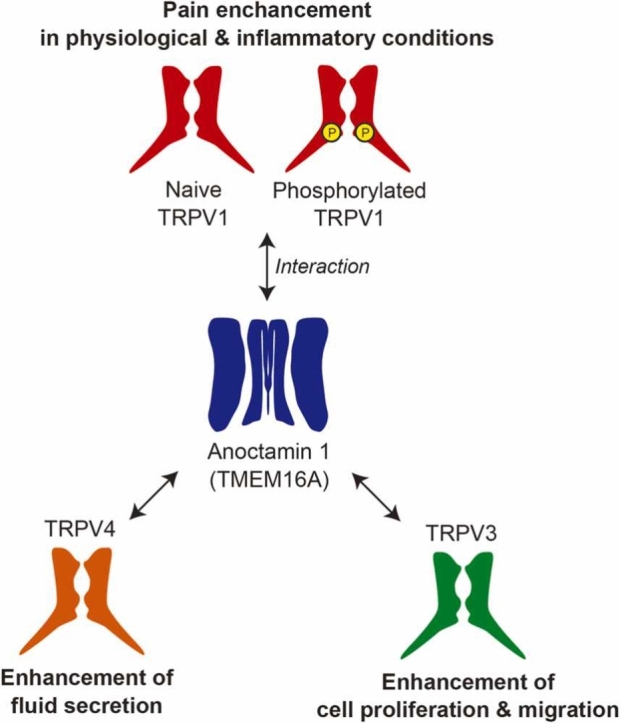

## Background

Transient receptor potential (TRP) channels, including TRP vanilloid (TRPV) 1, 2, 3, and 4; TRP melastatin (TRPM) 2, 3, 4, 5, and 8; TRP ankyrin (TRPA) 1; and TRP canonical (TRPC) 5 can be activated by temperature changes, according to previous reports (TRPV1–V4, TRPM8, TRPA1: [1], [2], TRPM2: [3], TRPM3: [4], TRPM4–M5: [5], TRPC5: [6]). These thermosensitive TRP channels are highly calcium-permeable, non-selective cation channels, with the exception of TRPM4 and TRPM5 [7]. Furthermore, the thermal responses of TRPV1, TRPA1 and TRPM8 have been observed in the planar lipid bilayer [8], [9], [10] and soybean proteoliposome [11]. This indicates that thermal responses could depend on the structure of ion channels, at least in these TRP channels. Although the structural biological mechanisms of thermosensitive TRP channels remain unclear, the heat-dependent structural changes in TRPV1 and TRPV3 has been analyzed using cryogenic electron microscopy (Cryo-EM) [12], [13]. In addition, the thermosensitivity of TRP channels is modulated by phosphorylation. TRPV1 is a major TRP channel showing decreases in activation threshold after phosphorylation by protein kinase C. Phosphorylated TRPV1 (pTRPV1) can be activated by temperatures over 35 °C [14], whereas unmodified TRPV1 functions as a noxious heat sensor, activating at temperatures over 43 °C [15]. The observed maximum currents induced by heat are smaller than those induced by chemical agonists including capsaicin [14], [16]. Calcium entering the cells through TRPV1 activation induces important cellular functions. For instance, cytosolic calcium binds to calcineurin in dendritic cells exposed to inflammatory situations including colitis, followed by dephosphorylation of the transcriptional factor nuclear factor of activated T cells (NFAT). Gene transcription via inflammatory cytokines including interleukin (IL)−6 is accelerated by the dephosphorylated NFAT that is translocated to the nucleus [17].

Cytosolic calcium also affects calcium-activated ion channels. Anoctamin 1 (ANO1, also called TMEM16A) is a major calcium-activated chloride channel identified in the mouse eye [18], *Xenopus* oocytes [19], and pancreatic cells [20]. In addition, the expression of ANO1 is increased by long-term stimulation with IL-4 in airway epithelial cells [20]. It was initially thought that ANO1 has eight transmembrane domains [18]; however Cryo-EM analysis has revealed 10 transmembrane domains in mouse ANO1 [21]. Mouse ANO1 can be weakly activated at basal concentrations of intracellular free calcium (100 nM) [22], and human ANO1 also has high sensitivity for free calcium, although it is slightly less sensitive than mouse ANO1 [22]. Namely, human ANO1 currents are not observed at a free intracellular calcium concentration of 100 nM. The intracellular calcium concentration should be the highest within several dozens of nanometers from the channel pore of calcium-permeable channels to ANO1 [23]. Thus, ANO1 can be strongly activated near calcium-permeable channels, such as TRP channels. Importantly, TRP channels physically interact with ANO1 (protein–protein interaction) and do not only have functional interactions (calcium increases through TRP channel activation enhances ANO1 activation). This review summarizes the latest understanding on the interactions between TRP channels and ANO1, focusing on thermosensitivity.

## TRPV1/ANO1 interaction in peripheral nervous system involved in burning pain sensation

TRPV1, expressed in primary sensory neurons involved in sensing burning pain, is a sensor that detects dangerous stimuli such as noxious heat over 43 °C [24]. ANO1 is also expressed in TRPV1-positive neurons and functions as another heat sensor. The activation threshold of ANO1 is approximately 44 °C, close to that of TRPV1. However, the Q10 value of ANO1 (approximately 20) is lower than that of TRPV1 (approximately 27) [25], [26]. Intriguingly, there is inflection point in heat-evoked ANO1 currents (two activation phases, slow and rapid increases in current amplitudes), and ANO1 currents rapidly inactivate after reaching their peak during noxious heat stimulation [25]. The temperature at which the inflection point occurs is thought to be the activation threshold for ANO1. Considering previous reports, it is evident that both TRPV1 and ANO1 are involved in noxious heat detection.

**Fig. 1:**
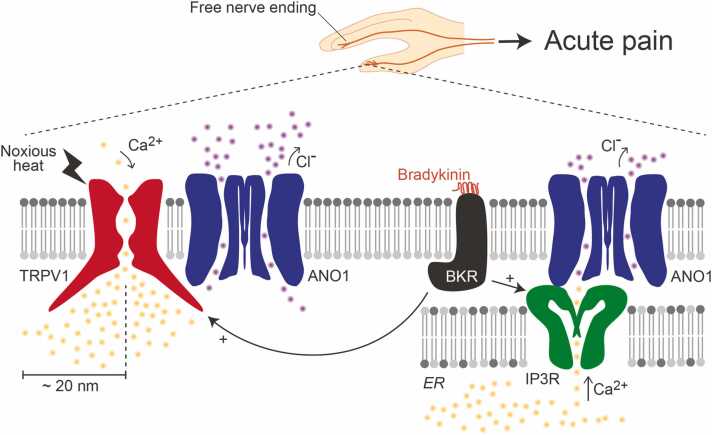
TRPV1/ANO1 interactions in free nerve endings contribute to acute pain sensation. Calcium entering cells through TRPV1, activated by noxious heat, strongly activates ANO1, bound to TRPV1. TRPV1 can also be activated by bradykinin receptor (BKR), which also activates inositol 1,4,5-triphosphate receptor (IP3R) on the endoplasmic reticulum (ER) membrane, and the released calcium activates ANO1 bound to IP3R. In primary sensory neurons, chloride channel activation, including ANO1, enhances neuronal excitation due to chloride efflux.

TRPV1 and ANO1 independently function as heat sensors; however, these channels functionally and physically interact. Because the intracellular chloride concentration is high in primary sensory neurons because they do not express the potassium–chloride cotransporter 2, which pumps out chloride [27]. Consequently, chloride efflux occurs in these neurons through the activation of anion channels. Regarding this chloride-dependent neuronal excitation, it has been reported that γ-aminobutyric acid evokes depolarization in rat dorsal root ganglia (DRG) neurons [28]. Importantly, noxious heat-evoked currents in cells expressing TRPV1 and ANO1 are significantly larger than those in cells expressing TRPV1 alone [16]. Thus, interactions between TRPV1 and ANO1 amplify TRPV1-mediated pain sensation, although TRPV1 alone can also induce neuronal excitation (Fig. 1).

Additionally, bradykinin-induced ANO1 currents are observed in the cell bodies of isolated DRG neurons [29]. Furthermore, ANO1 conditional knockout mice showed significantly reduced bradykinin-evoked pain-related behavior over a 30-min period [30]. While the role and presence of the endoplasmic reticulum (ER) in free nerve endings remain unclear, high concentrations of bradykinin induce pain sensations within 1 min, independent of TRPV1 activation [31]. ANO1 is located close to inositol 1,4,5-triphosphate (IP3) receptors on the ER membrane, and calcium released from the ER via IP3 receptors activates ANO1 in the plasma membrane [32], [33]. This protein–protein interaction between the IP3 receptor and ANO1 has been identified using immunoprecipitation assays, proximity ligation assays, and stochastic optical reconstruction microscopy. Thus, ANO1 expressed in primary sensory neurons could be activated by two pathways: TRPV1/ANO1 and IP3 receptor/ANO1 axis (Fig. 1).

**Fig. 2:**
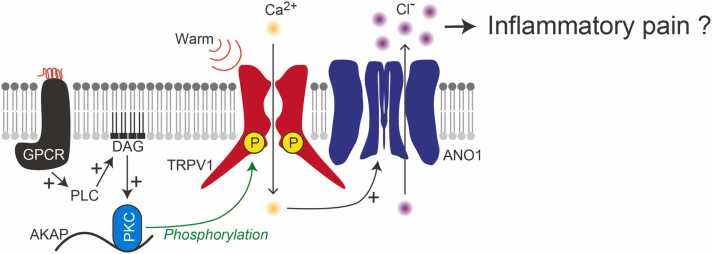
Phosphorylated TRPV1/ANO1 interactions in inflammatory pain. Downstream of G protein-coupled receptor (GPCR), phospholipase C (PLC) is activated, followed by the generation of diacylglycerol (DAG) derived from phospholipids from the plasma membrane. DAG activates protein kinase C (PKC), forming a complex with A-kinase anchoring protein (AKAP), and the activated PKC phosphorylates TRPV1. After phosphorylation, TRPV1 can be activated by warm temperatures, such as body temperature (37 °C), and the weak calcium influx evoked by TRPV1 opening strongly activates ANO1, followed by the enhancement of pain signals.

Interactions between TRPV1 and ANO1 could be also involved in inflammatory pain sensations (Fig. 2). G protein-coupled receptors such as the bradykinin receptor are activated in inflammation [34]. In inflammatory conditions, TRPV1 is phosphorylated at the Ser800 residue by protein kinase C via A-kinase anchoring protein, which binds to the TRPV1 C-terminus [35], [36]. pTRPV1 can be activated by temperatures over 35 °C [14], and it can interact with ANO1 at lower temperatures than unphosphorylated TRPV1. Therefore, innocuous stimuli can evoke TRPV1/ANO1 interactions. After application of phorbol 12-myristate 13-acetate, a protein kinase C activator, functional interactions between pTRPV1 and ANO1 were induced at 37 °C in HEK293T cells expressing TRPV1 and ANO1 [16]. Although pTRPV1/ANO1 interactions have yet to be confirmed in vivo, this interaction could contribute to the amplification of inflammatory pain signals in primary sensory neurons (Fig. 2).

Additional investigations into TRP/ANO1 interactions focusing on other TRP channels except TRPV1 in primary sensory neurons are necessary. TRPV2 (also called VRL-1) is also expressed in DRG neurons and can be activated by noxious heat over 52 °C [37]. However, the involvement of TRPV2 in thermal sensations were not observed using pain-related behavioral tests, such as the hot plate test, in conditional TRPV2 knockout mice [38]. Furthermore, a recent report suggested that the burning pain sensations could be evoked by the combination of three TRP channels including TRPM3, TRPV1, and TRPA1 [39]. In addition, the involvement of TRPM2 in warmth sensing has been suspected [40]. Although these reports remain controversial [41], [42], there are possibilities that other TRP channels also interact with ANO1 in primary sensory neurons.

## TRPV3/ANO1 interactions in keratinocytes

**Fig. 3:**
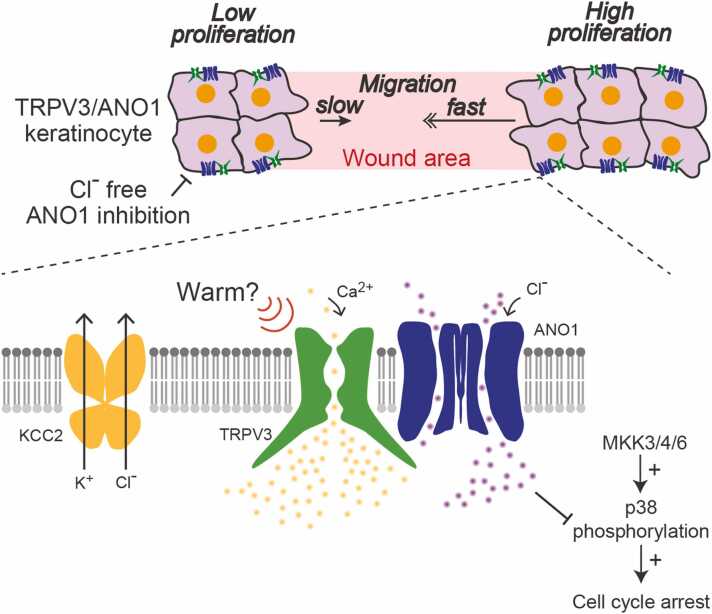
Summary of cell proliferation and migration controlled by TRPV3/ANO1 interactions. In human skin keratinocytes, TRPV3/ANO1 interactions accelerate keratinocyte proliferation and migration. Potassium-chloride cotransporter 2 (KCC2) continuously pumps out chloride from intracellular region. TRPV3/ANO1 interaction occurs chloride influx, and this event inhibit p38 phosphorylation induced by mitogen-activated protein kinase kinase (MKK) 3/4/6 activation. Phosphorylated p38 promotes cell cycle arrest. TRPV3/ANO1 interaction accelerates cell proliferation and likely enhances wound healing.

Skin keratinocytes functionally express thermosensitive TRP channels including TRPV3 and TRPV4. These TRP channels are warm-sensitive according to previous reports [43], [44], [45]. Although ANO1 expression in mouse keratinocytes is not existent or quite low [46], ANO1 mRNA and protein have been detected in normal human epidermal keratinocytes (NHEKs) [47]. Although TRPV4/ANO1 interactions remain undetermined in NHEKs, TRPV3/ANO1 interactions could be involved in physiological mechanisms of human keratinocytes. Functional interactions between TRPV3 and ANO1 have been suggested using the TRPV3 agonist camphor with the specific ANO1 inhibitor Ani9 [47]. The camphor-elicited currents in NHEKs were dependent on extracellular calcium, and the chloride-dependent equilibrium potential was shifted to a higher voltage when using extracellular solution containing low concentrations of chloride. Furthermore, using the ANO1 inhibitor or extracellular chloride-free medium suppressed NHEK migration and proliferation, mediated by the mitogen-activated protein kinase (MAPK) cascade [47]. Notably, ANO1 inhibition specifically increased the proportion of cells in the G0/G1 phase of the cell cycle. These properties could be involved in wound healing (Fig. 3).

## TRPV4/ANO1 interactions in fluid secretions

TRPV4 (also called OTRPC4, VRL-2, VR-OAC, and TRP12) is also co-expressed with ANO1 in some secretory tissues, including the choroid plexus, salivary glands, lacrimal glands, and sweat glands [48]. TRPV4 had been identified as an osmosensor [49]; however, it has also been reported that an arachidonic acid metabolite, 5',6'-epoxyeicosatrienoic acid, which is generated via plasma membrane stretch, directly activates TRPV4 [50]. Furthermore, TRPV4 can be activated by warm temperatures [51], [52]. Dual stimulation by warmth and hypotonicity strongly activates TRPV4 [53]. TRPV4/ANO1 interactions were first reported in a study of cerebrospinal fluid secretion [54]. This study suggested that ANO4, ANO6, and ANO10 cannot functionally interact with TRPV4. Importantly, the chloride flux caused by ANO1 activation can drive water movement in the same direction as ion flow across the cell membrane [54]. Thus, fluid secretion is increased when TRPV4 interacts with ANO1, followed by chloride efflux at resting potential in secretory cells.

It has been known that the autonomic nervous system is strongly involved in saliva secretion. Specifically, IP3 receptor types 2 and 3 are major factors in this process. Double-knockout mice without both receptors exhibit impairments in normal saliva excretion as well as that induced by pilocarpine, an acetylcholine receptor agonist [55]. Moreover, the IP3 pathway, induced downstream of acetylcholine receptors, is also involved in ANO1 activation localized to the apical plasma membrane of acinar cells [18], [19], [56], [57]. Importantly, it has been suggested using whole-cell patch-clamp recording and proximity ligation assays that TRPV4/ANO1 interactions support the fluid secretions [32]. Namely, there could be fluid secretion mechanism independent of the autonomic nervous system.

**Fig. 4:**
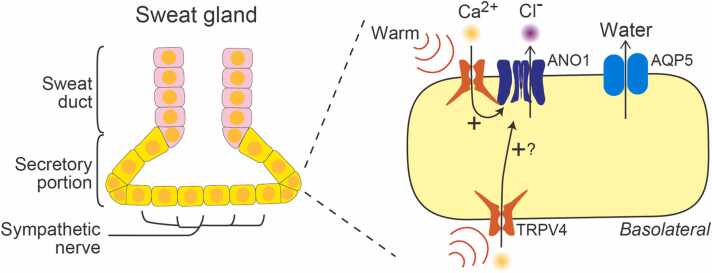
TRPV4/ANO1 interactions in sweat glands. Sweat glands are composed of sweat ducts and secretory tissue. While TRPV4 is expressed in both areas, ANO1 is localized to the apical membrane of secretory cells. TRPV4/ANO1 interactions could be independent of the sympathetic nervous system. The local increases in skin temperature can activate TRPV4 channels, activating ANO1 and causing a chloride efflux toward the glandular cavity, followed by water efflux through aquaporin 5 (AQP5). Although TRPV4 is also expressed in the basolateral membrane of the secretory cells, it is unclear whether calcium entering cells through the activation of basolateral TRPV4 activates ANO1 localized to the apical membrane.

In fact, a recent report suggested that the fluid secretion resulting from TRPV4/ANO1 interactions involves independent molecular mechanisms from the autonomic nervous system. Warming accelerates the fluid secretion in sweat glands [46]. Sweating without acetylcholine at an environmental temperate of 35 °C was inhibited in TRPV4-deficient mice and by topical application of the selective ANO1 antagonist Ani9 [46]. The real skin temperature at an environmental temperature of 35 °C and the effects of Ani9 on bestrophin-2, which is also involved in sweating [58], remain unknown. Therefore, although additional investigations are needed, TRPV4/ANO1 interactions could enhance sweating corresponding to increases in environmental temperature (Fig. 4).

## Conclusions

As summarized in this review, interactions between ANO1 and TRPV1, TRPV3, and TRPV4 are dependent on temperature and involved in physiological functions, including pain sensation, wound healing, and fluid secretion, respectively. However, there is a need for further investigations into the binding sites between TRP channels and ANO1. Furthermore, differences between human and mouse keratinocytes regarding TRPV3/ANO1 interactions remain unclear. Additional studies using ANO1 conditional knockout animals are also needed. Information on other types of TRP/ANO1 interactions is still needed. For instance, there is possibility that TRPV1, TRPV2, TRPV4, TRPM2, and TRPM3 in pancreatic β-cells interact with ANO1 following insulin secretion (TRPV1: [59], TRPV2: [60], TRPV4: [61], TRPM2: [3], TRPM3: [62], ANO1: [63]). Many reports have been published separately on TRP channels and ANO1; however, research should also focus on interactions with ANO1 to clarify the related mechanisms and identify potential therapeutic targets.

## Funding

This work was supported by the 10.13039/100007449Takeda Science Foundation.

## CRediT authorship contribution statement

**Yasunori Takayama:** Writing – review & editing, Writing – original draft, Visualization, Funding acquisition.
